# Puberty timing and markers of cardiovascular structure and function at 25 years: a prospective cohort study

**DOI:** 10.1186/s12916-021-01949-y

**Published:** 2021-03-25

**Authors:** Gillian M. Maher, Lisa Ryan, Fergus P. McCarthy, Alun Hughes, Chloe Park, Abigail Fraser, Laura D. Howe, Patricia M. Kearney, Linda M. O’Keeffe

**Affiliations:** 1 INFANT Research Centre, University College Cork, Cork, Ireland; 2 School of Public Health, University College Cork, Cork, Ireland; 3 Department of Obstetrics and Gynaecology, University College Cork, Cork, Ireland; 4 Department of Population Science and Experimental Medicine, Institute of Cardiovascular Science, Faculty of Population Health Sciences, University College London, Gower Street, London, WC1E 6BT UK; 5 MRC Unit for Lifelong Health and Ageing at University College London, London, UK; 6 Population Health Sciences, Bristol Medical School, Oakfield House, Oakfield Grove, Bristol, BS82BN UK

**Keywords:** Puberty, Cardiovascular structure, ALSPAC

## Abstract

**Background:**

Whether earlier onset of puberty is associated with higher cardiovascular risk in early adulthood is not well understood. Our objective was to examine the association between puberty timing and markers of cardiovascular structure and function at age 25 years.

**Methods:**

We conducted a prospective birth cohort study using data from the Avon Longitudinal Study of Parents and Children (ALSPAC). Participants were born between April 1, 1991, and December 31, 1992. Exposure of interest was age at peak height velocity (aPHV), an objective and validated growth-based measure of puberty onset. Outcome measures included cardiovascular structure and function at age 25 years: carotid intima-media thickness (CIMT), left ventricular mass index (LVMI) and relative wall thickness (RWT), pulse wave velocity (PWV) and systolic blood pressure (SBP). Multiple imputation was used to impute missing data on covariates and outcomes. Linear regression was used to examine the association between aPHV and each measure of cardiac structure and function, adjusting for maternal age, gestational age, household social class, maternal education, mother’s partner’s education, breastfeeding, parity, birthweight, maternal body mass index, maternal marital status, maternal prenatal smoking status and height and fat mass at age 9. All analyses were stratified by sex.

**Results:**

A total of 2752–4571 participants were included in the imputed analyses. A 1-year older aPHV was not strongly associated with markers of cardiac structure and function in males and females at 25 years and most results spanned the null value. In adjusted analyses, a 1-year older aPHV was associated with 0.003 mm (95% confidence interval (CI) 0.00001, 0.006) and 0.0008 mm (95% CI − 0.002, 0.003) higher CIMT; 0.02 m/s (95% CI − 0.05, 0.09) and 0.02 m/s (95% CI − 0.04, 0.09) higher PWV; and 0.003 mmHg (95% CI − 0.60, 0.60) and 0.13 mmHg (95% CI − 0.44, 0.70) higher SBP, among males and females, respectively. A 1-year older aPHV was associated with − 0.55 g/m^2.7^ (95% CI − 0.03, − 1.08) and − 0.89 g/m^2.7^ (95% CI − 0.45, − 1.34) lower LVMI and − 0.001 (95% CI − 0.006, 0.002) and − 0.002 (95% CI − 0.006, 0.002) lower RWT among males and females.

**Conclusions:**

Earlier puberty is unlikely to have a major impact on pre-clinical cardiovascular risk in early adulthood.

**Supplementary Information:**

The online version contains supplementary material available at 10.1186/s12916-021-01949-y.

## Background

Cardiovascular disease (CVD) is a major cause of morbidity and mortality worldwide, with 7.8 million premature CVD deaths estimated in 2025 if current trajectories are not altered [1, 2]. CVD risk originates in early life and tracks through the life course [3, 4]. Onset of puberty is a transitional period between childhood and adulthood with intense hormonal activity, including the release of gonadotropins, leptin, sex-steroids and growth hormone, leading to physical bodily changes and the appearance of secondary sexual characteristics. The most striking feature of puberty is a spurt in height which occurs in males in late puberty and is highly correlated with secondary sexual characteristics such as enlargement of larynx, deepening of voice and genitalia development [5]. Conversely, growth spurts tend to start earlier in girls, often coinciding with breast development and menarche, but with shorter duration in comparison to boys [6–9]. Age at puberty onset has been decreasing for several decades, with increasing childhood adiposity (a condition of being severely overweight, or obese) thought to play a substantial role [10].

Several studies to date have examined the association between earlier puberty timing and CVD risk, with conflicting findings [11–17]. A key limitation of many studies including observational cohort studies and Mendelian randomisation (MR) designs has included lack of adjustment for early childhood adiposity or use of indirect measures of adiposity such as BMI for adjustment, resulting in residual confounding of the puberty timing-CVD risk associations by early life adiposity [11, 12, 14, 15, 18, 19]. In addition, though age at menarche offers a reliable marker of puberty timing in females, most previous studies have assessed puberty timing using self-reported measures among males such as voice change, facial hair and pubic hair [20, 21], leading to measurement error [22]. Thus, studies examining the association between objectively measured puberty timing and CVD risk in males and females, while adjusting for direct measures of childhood adiposity are required to better understand the aetiology of puberty timing and CVD risk in early life.

Using the objective growth-based measure of puberty onset (age at peak height velocity [aPHV]), we aimed to better understand the association between puberty timing and pre-clinical cardiovascular risk in early adulthood using markers of cardiovascular structure and function at age 25 years (carotid intima-media thickness, left ventricular mass index and relative wall thickness, pulse wave velocity and systolic blood pressure).

## Methods

### Study participants

We used data from the Avon Longitudinal Study of Parents and Children (ALSPAC), a prospective birth cohort study based in Southwest England [23–25]. The women invited to participate in this study were pregnant with an expected delivery date between April 1, 1991, and December 31, 1992, and living in one of the three Bristol-based health districts. A detailed description of this study is available elsewhere [23–25].

The initial number of pregnancies enrolled was 14,541. When the oldest children were approximately 7 years of age, an attempt was made to increase the initial sample with eligible children who did not join the study originally. This resulted in an additional 913 children being enrolled. Therefore, the total sample size was 15,454 pregnancies. Of these 14,901 were alive at 1 year of age. In the three decades since enrolment, ALSPAC has used questionnaires completed by both parents and children, routine medical data and research clinics as methods of follow-up. The clinics took place when the participants were 7, 9, 10, 11, 13, 15, 17 and 25 years old [23–27].

Ethical approval for the ALSPAC study was obtained from the ALSPAC Law and Ethics Committee and Local Research Ethics Committees. Informed consent for the use of data collected via questionnaires and clinics was obtained from participants following the recommendations of the ALSPAC Law and Ethics Committee at the time. The study website contains details of all the data that is available through a fully searchable data dictionary and variable search tool: http://www.bristol.ac.uk/alspac/researchers/our-data/ [28].

### Exposure

#### Assessment of timing of puberty

Puberty is a period of intense hormonal activity and rapid growth, of which the most striking feature is the spurt in height [6]. aPHV is a validated measure of puberty timing [6] captured using Superimposition by Translation and Rotation (SITAR), a non-linear multilevel model with natural cubic splines which estimates the population average growth curve and departures from it as random effects [29, 30]. Using SITAR, PHV was identified in ALSPAC participants using numerical differentiation of the individually predicted growth curves, with aPHV being the age at which the maximum velocity is observed [29–31]. Repeated height data included measurements from research clinics and were used here to derive aPHV using SITAR. Individuals with at least one measurement of height from 5 years to < 10 years, 10 years to < 15 years and 15 years to 20 years were included. Data were analysed for males and females separately. The model was fitted using the SITAR package in R version 3.4.1, as described elsewhere [31]. Further details on how aPHV was derived are described elsewhere [31, 32].

### Outcomes

#### Assessment of cardiovascular risk

Carotid intima-media thickness, left ventricular mass index and relative wall thickness, pulse wave velocity and systolic blood pressure were measured at research clinics at age 25 years. Participants fasted for 6 h before the clinic, with the exception of those participants with a diagnosis of diabetes or a condition that would not allow fasting. Carotid intima-media thickness scans of the left and right common carotid arteries were performed using a CardioHealth Panasonic system with a 13–5 MHz linear array broadband transducer according to a standardised protocol. Carotid intima-media thickness has been validated in several studies as a strong predictor of CVD-risk [33–35]. Participants lay on a couch with their arms by their side, while a trained researcher performed the ultrasound test on both sides of their neck. Right and left carotid intima-media thickness measurements were taken to be the average of 3 end-diastolic measurements of the far-wall of the common carotid artery over a length of 5–10 mm, and 10 mm adjoining the bifurcation. The mean of both right and left carotid intima-media thickness measures was calculated and used here.

Echocardiography was performed by two experienced echo-cardiographers using a Philips EPIQ 7G Ultrasound System equipped with a X5-1 transducer in accordance with American Society of Echocardiography guidelines; these techniques are described elsewhere [36]. Left ventricular mass, assessed by ultrasound, was indexed to height^2.7^ to adjust for body surface area. Relative wall thickness was calculated using left ventricular internal diameter in diastole and thickness of the left ventricular posterior wall and septal wall. Pulse wave velocity was measured using a Vicorder device (Skidmore Medical, Bristol, UK) at femoral and carotid artery level which has been validated in previous studies in adolescents [37]. Ten pulse wave velocity measurements were taken within ≤ 0.5 m/s of each other. These were averaged to give a measurement of arterial stiffness. Systolic blood pressure was measured (typically from the right arm) with the subject in a sitting position using an Omron 705-IT machine [36].

### Covariates

We selected potential confounders a priori and used a directed acyclic graph (DAG) to encode this causal knowledge of this research question. In summary, we have included only covariates in our model, which we believe to be common causes of the exposure and outcome and have excluded any variables that might be potential mediators of the association [38]. Therefore, we considered the following as possible confounders of the association between aPHV and cardiovascular structure and function at age 25 years; maternal age, gestational age at birth, household social class, maternal education, mother’s partner’s education, breastfeeding of baby until 3 months, parity, birthweight, maternal body mass index (BMI), maternal marital status, maternal smoking status during first 3 months of pregnancy, and height and DXA-determined fat mass at age 9.

Maternal age was reported in the mother’s antenatal questionnaires. Gestational age at birth was estimated from clinical records. Household social class was measured as the highest of the mother’s or her partner’s occupational social class using data on job title and details of occupation collected about the mother and her partner from the mother’s questionnaire at 32 weeks gestation. Social class was derived using the standard occupational classification (SOC) codes developed by the UK Office of Population Census and Surveys and classified as I professional, II managerial and technical, IIINM non-manual, IIIM manual and IV&V part skilled occupations and unskilled occupations.

A questionnaire at 32 weeks gestation asked mothers to report on educational attainment, which was categorised as below O-Level (Ordinary Level; exams taken in different subjects usually at age 15–16 at the completion of legally required school attendance, equivalent to today’s UK General Certificate of Secondary Education), O-Level only, A-Level (Advanced-Level; exams taken in different subjects usually at age 18) or university degree or above.

Breastfeeding information was collected via questionnaires administered at 4 weeks, 6 months and 15 months. Parity was defined as the number of previous pregnancies that had resulted in a live or stillborn infant collected at 18 weeks gestation. Birthweight was extracted from medical records. Maternal height and weight data were self-reported from a questionnaire administered at 12 weeks gestation; these were used to calculate maternal BMI. Marital status was obtained from antenatal questionnaires and classified as never married, married and widowed, divorced, or separated. Smoking in the first trimester of pregnancy was self-reported by mothers at 18 weeks gestation; responses to smoking any tobacco (cigarettes, cigars, pipes, or other) were grouped as follows: no smoking, < 10 per day, 10–19 per day or greater than 19 per day, and were re-categorised as a dichotomous variable (smoking: yes/no).

Height and fat mass of offspring was measured at clinics at age 9 years. Standing height was measured to the last complete mm using the Harpenden Stadiometer. Fat mass (in kg, less head) was derived from whole body DXA scans performed using a GE Lunar Prodigy (Madison, WI, USA) narrow fan beam densitometer.

### Statistical analysis

Statistical analysis was performed using Stata MP 14.2. Linearity of association between aPHV and cardiovascular structure and function in males and females was assessed by comparing fit of models regressing carotid intima-media thickness, left ventricular mass index and relative wall thickness, pulse wave velocity, and systolic blood pressure on fourths of aPHV (treated as a continuous exposure) to models regressing carotid intima-media thickness, left ventricular mass index and relative wall thickness, pulse wave velocity and systolic blood pressure on fourths of aPHV (treated as a categorical exposure); models were then formally compared using a likelihood ratio test. Data on aPHV and all cardiovascular outcomes were normally distributed. We used linear regression to examine the association between aPHV and each measure of cardiac structure and function. All analyses were stratified by sex to examine whether associations of aPHV and each outcome differed for males and females.

#### Dealing with missing data

There were 1197–2193 participants with complete data for aPHV, each outcome and all covariates. Cardiac structure and function were also measured at the 18 year ALSPAC clinic. To increase efficiency and minimise selection bias, we used multivariate multiple imputation to impute missing data on covariates and outcomes in all participants that had a measure of aPHV and had a measure of the outcome at the age 18 year or age 25 year research clinic (*N* = 4339 for carotid intima-media thickness, *N* = 2752 for left ventricular mass index, *N* = 2776 for relative wall thickness, *N* = 3964 for pulse wave velocity and *N* = 4571 for systolic blood pressure). We carried out 20 cycles of regression switching and generated 20 imputation datasets [39]. We then examined associations of aPHV and outcomes in these multiple imputed datasets; results are averaged across the results from each of these 20 datasets using Rubin’s rules, taking account of uncertainty in the imputation so that the standard errors for any regression coefficients (used to calculate 95% confidence intervals) take account of uncertainty in the imputations and uncertainty in the estimate [39]. See Additional file 1: Table S1 for a list of the variables included in the multiple imputation models and how they were entered into the models.

We repeated the main adjusted analysis in the observed dataset, among participants with complete-case data on exposure, outcome and covariates (*N* = 1199 for carotid intima-media thickness, *N* = 1197 for left ventricular mass index, *N* = 1203 for relative wall thickness, *N* = 1394 for pulse wave velocity and *N* = 2193 for systolic blood pressure).

## Results

The distribution/proportions of baseline characteristics by fourths of age at peak height velocity are shown in Table 1, and the characteristics of the cohort included in the analysis (by sex) are shown in Additional file 1: Table S2. A total of 2752–4571 participants were included in the imputed analyses. The mean aPHV was 13.54 years in males and 11.73 years in females. Findings from linearity tests of the association between aPHV and cardiac structure and function demonstrated little evidence of departures from linearity, allowing aPHV to be examined as a continuous exposure (Additional file 1: Table S3). The distribution of variables in observed and imputed data is shown in Additional file 1: Tables S4-S8; distributions of most variables were broadly similar between observed and imputed datasets with some minor differences only for socio-economic position indicators which were slightly higher in the observed datasets.

**Table 1:** Distribution/proportions of baseline characteristics by fourths of age at peak height velocity based on imputed data

	**Quartile 1,** ***n*** **(%)**	**Quartile 2,** ***n*** **(%)**	**Quartile 3,** ***n*** **(%)**	**Quartile 4,** ***n*** **(%)**
**Household social class**				
Professional	182 (16.78)	188 (17.38)	218 (20.02)	213 (19.67)
Managerial/technical	501 (46.18)	479 (44.09)	517 (47.64)	514 (47.45)
Non-manual	248 (22.87)	273 (25.19)	230 (21.22)	235 (21.75)
Manual/part skills/unskilled	154 (14.17)	145 (13.34)	121 (11.12)	121 (11.13)
**Maternal education**	182 (16.78)	188 (17.38)	218 (20.02)	213 (19.67)
Less than O level	217 (20.03)	181 (16.72)	164 (15.16)	197 (18.18)
O level	372 (34.24)	392 (36.02)	368 (33.83)	330 (30.51)
A level	298 (27.49)	293 (27.04)	339 (31.20)	323 (29.80)
Degree or above	198 (18.24)	219 (20.22)	215 (19.81)	233 (21.51)
**Mother’s partner’s education**				
Less than O level	312 (28.73)	261 (24.14)	246 (22.64)	237 (21.87)
O level	230 (21.19)	232 (21.39)	223 (20.52)	250 (23.07)
A level	300 (27.65)	331 (30.51)	321 (29.56)	300 (27.66)
Degree or above	243 (22.43)	260 (23.96)	296 (27.28)	297 (27.40)
**Breastfeeding until 3 months**				
Exclusively	389 (35.83)	407 (37.53)	408 (37.59)	383 (35.35)
Non-exclusively	523 (48.12)	514 (47.37)	555 (51.12)	570 (52.61)
Never	174 (16.05)	164 (15.10)	122 (11.29)	130 (12.04)
**First-born child**	559 (51.51)	506 (46.67)	547 (50.42)	516 (47.64)
**Maternal marital status**				
Never married	143 (13.14)	139 (12.79)	127 (11.74)	144 (13.29)
Married	883 (81.42)	896 (82.60)	913 (84.01)	891 (82.28)
Widowed/divorced/separated	59 (5.44)	50 (4.61)	46 (4.25)	48 (4.43)
**Maternal smoking status**				
No	892 (82.20)	930 (85.68)	931 (85.82)	924 (85.28)
Yes	193 (17.80)	155 (14.32)	154 (14.18)	159 (14.72)
	**Mean (SE)**	**Mean (SE)**	**Mean (SE)**	**Mean (SE)**
Maternal age at delivery (years)	29.25 (0.14)	29.35 (0.14)	29.64 (0.13)	29.74 (0.14)
Gestational age (weeks)	39.54 (0.05)	39.48 (0.05)	39.32 (0.05)	39.37 (0.05)
Birthweight (kg)	3.35 (0.01)	3.41 (0.01)	3.44 (0.01)	3.50 (0.01)
Maternal BMI (kg/m^2^)	23.13 (0.11)	22.80 (0.11)	22.63 (0.11)	22.70 (0.11)
Height at age 9 (cm)	141.36 (0.19)	138.95 (0.19)	139.12 (0.18)	138.29 (0.18)
Fat mass at age 9 (kg)	37.52 (0.25)	33.69 (0.21)	33.22 (0.20)	31.87 (0.18)

*Abbreviations: SE* standard error, *BMI* body mass index

### Age at peak height velocity and cardiovascular structure and function

#### Males

There was little evidence of association between aPHV and cardiac structure and function in males, with results mostly spanning the null value. For example, in confounder-adjusted analyses, a one-year older aPHV was associated with 0.003 mm (95% confidence interval (CI) 0.00001, 0.006) higher carotid intima-media thickness, 0.02 m/s (95% CI − 0.05, 0.09) higher pulse wave velocity and 0.003 mmHg (95% CI − 0.60, 0.60) higher systolic blood pressure at 25 years. A 1-year older aPHV was associated with a − 0.55 g/m^2.7^ (95% CI − 0.03, − 1.08) lower left ventricular mass index and − 0.001 (95% CI − 0.006, 0.002) lower relative wall thickness at 25 years (Table 2).

**Table 2:** Associations between age at peak height velocity and cardiac structure and function in males and females at age 25

	Males		Females	
Outcome	Crude estimate (95% CI)^**a**^	Adjusted estimate (95% CI)^**a,b**^	Crude estimate (95% CI)^**a**^	Adjusted estimate (95% CI)^**a,b**^
**CIMT**	0.0001 (− 0.002, 0.003)	0.003 (0.00001, 0.006)	− 0.0007 (− 0.003, 0.002)	0.0008 (− 0.002, 0.003)
**LVMI**	− 1.00 (− 0.50, − 1.50)	− 0.55 (− 0.03, − 1.08)	− 1.17 (− 0.74, − 1.59)	− 0.89 (− 0.45, −1.34)
**RWT**	− 0.003 (− 0.007, 0.0009)	− 0.001 (− 0.006, 0.002)	− 0.002 (− 0.006, 0.001)	− 0.002 (− 0.006, 0.002)
**PWV**	− 0.04 (− 0.11, 0.02)	0.02 (− 0.05, 0.09)	− 0.03 (− 0.09, 0.01)	0.02 (− 0.04, 0.09)
**SBP**	− 0.97 (− 0.40, − 1.54)	0.003 (− 0.60, 0.60)	− 1.20 (− 0.69, − 1.71)	0.13 (− 0.44, 0.70)

*aPHV* age at peak height velocity, *CI* confidence interval, *CIMT* carotid intima-media thickness, *LVMI* left ventricular mass index, *RWT* relative wall thickness, *PWV* pulse wave velocity, *SBP* systolic blood pressure

^a^Association of a one-year older aPHV with each outcome

^b^Adjusted for maternal age, gestational age, household social class, maternal education, mother’s partner’s education, breastfeeding of baby until 3 months, parity, birthweight, maternal body mass index, maternal marital status, maternal smoking status during first 3 months of pregnancy, and height and fat mass of offspring at age 9

#### Females

Similar to males, there was little evidence of association observed between aPHV and cardiac structure and function in females, with results mostly spanning the null value. In confounder-adjusted analyses, a one-year older aPHV was associated with 0.0008 mm (95% CI − 0.002, 0.003) higher carotid intima-media thickness, 0.02 m/s (95% CI − 0.04, 0.09) higher pulse wave velocity and 0.13 mmHg (95% CI − 0.44, 0.70) higher systolic blood pressure at 25 years. A one-year older aPHV was associated with a − 0.89 g/m^2.7^ (95% CI − 0.45, − 1.34) lower left ventricular mass index and − 0.002 (95% CI − 0.006, 0.002) lower relative wall thickness at 25 years (Table 2).

Unadjusted and confounder adjusted results were not materially different for each outcome in males and females (Table 2).

Adjusted associations of aPHV with measures of cardiac structure and function among participants with complete-case data on exposure, outcome and covariates were comparable to the main results obtained using imputed datasets (Additional file 1: Table S9).

## Discussion

This study aimed to better understand the association between puberty timing and cardiovascular structure and function at age 25 years using aPHV as an objective growth-based measure of puberty onset and carotid intima-media thickness, left ventricular mass index and relative wall thickness, pulse wave velocity and systolic blood pressure as pre-clinical measures of cardiovascular risk. There was little evidence of association between aPHV and cardiovascular structure and function at 25 years, with results spanning the null in all but left ventricular mass index for which the association was inverse.

Previous studies have shown that a ∼ 5 g/m^2^ higher left ventricular mass index can be predicted to correspond to a 7–20% increase in CVD morbidity and mortality [40–42]. Given the small differences in left ventricular mass index per year older aPHV found here, our findings suggest that earlier puberty is unlikely to have a major impact on pre-clinical cardiovascular risk in early adulthood.

### Comparison with other studies

Several studies to date have examined the association between puberty timing and clinical endpoints such as CVD and traditional cardiovascular risk factors such as blood pressure and adiposity, with conflicting findings [11–13, 18, 20, 21, 43]. However, these studies used different measures of puberty timing (objective vs. self-report) [43, 44] and had varying degrees of adjustment for confounding (particularly early childhood adiposity) [11–13, 18] and different ranges in follow-up and age of measurement of outcomes [17, 21, 45].

In contrast, fewer studies have examined associations of puberty timing with measures of cardiac structure and function, which are used clinically to assess arterial stiffness, left ventricular hypertrophy and cardiac remodelling [44]. Hardy and colleagues found evidence of an association between later puberty timing (measured using age at menarche) and lower left ventricular mass and relative wall thickness in females in the National Study of Health and Development (NSHD) (*N* = 1385), though results attenuated after adjustment for childhood or adult adiposity [44]. In contrast, there was no strong evidence of an association between puberty timing (measured via physical examination) and any measure of cardiac structure or function in males in the NSHD, a finding comparable to ours [44]. Our findings are also comparable to results from the Young Finns Cohort (*N* = 794) which demonstrated no strong evidence of associations between puberty timing (measured via age at menarche) and carotid intima-media thickness in females at age 30–39 years [17]. Similarly, in a study of 800 women aged 50 to 81 years in Southern Germany, age at menarche was not associated with carotid intima-media thickness in both unadjusted and confounder-adjusted analyses [45].

### Strengths and limitations

Strengths of this study include use of an objective measure of puberty timing (aPHV) based on prospective, repeated measures of height from age 5 years to 20 years which is a more accurate marker of puberty timing than self-reported measures. We also used measures of pre-clinical CVD, which strongly predict later cardiovascular risk. We adjusted for childhood adiposity using DXA fat mass at age 9, allowing us to adjust for directly measured pre-pubertal adiposity, a key limitation in several previous studies [11–13, 18]. However, there are also several limitations; a limitation of our adiposity adjustment may include the possibility that DXA fat mass is measured after pubertal onset for a small proportion of participants (*N* = 1 for males, *N* = 41 for females) giving rise to the possibility of collider bias if DXA fat mass at age 9 is a mediator of the puberty timing-cardiac risk association. However, we believe that any bias introduced due to this is minimal, given that prior work in this cohort has shown that puberty has little impact on post-pubertal fat mass gain [32] (suggesting fat mass is an unlikely mediator of any associations of puberty timing with health outcomes) and that analyses without adjustment for fat mass were similar to confounder-adjusted results. Nevertheless, though we have shown that our approach to adjustment for adiposity here is not likely to have introduced bias to our results, the direction of causality of the relationship between adiposity and puberty timing is complex given the potential shared genetic architecture between the traits. In addition, the outcome measures used may be subject to measurement error which may have led to biased estimates. However, any measurement error is likely to be non-differential and would therefore bias our results towards to the null [46, 47]. Further limitations include potential for selection bias due to missing data and attrition from the cohort. However, the use of multivariate multiple imputation aimed to minimise bias and a loss of statistical power by imputing missing covariate and outcome data, and results in multiply imputed and complete-case data were similar. However, replication of findings in studies with larger sample sizes may be needed in order to provide more precise estimates of associations. Moreover, our outcomes were measured at age 25 years, and while the pre-clinical measures of CVD used here are strong predictors of later cardiovascular risk [33, 48], young adulthood might be too early to detect changes in cardiac structure and function that progress with age. Therefore, future studies examining a puberty timing and CVD risk relationship in older populations may be worthwhile. Finally, the vast majority of the ALSPAC cohort are of White ethnicity [23], and a key limitation of our study is the generalisability of the findings to non-White ethnicities.

## Conclusion

Age at peak height velocity was not strongly associated with measures of cardiac structure and function among males and females at age 25 years. Earlier puberty is unlikely to have a major impact on pre-clinical cardiovascular risk in early adulthood.

## Supplementary Information

**Additional file 1: Table S1.** [Variables used in multivariable multiple imputation models]. **Table S2.** [Characteristics of ALSPAC participants included in the analysis by sex and based on imputed data]. **Table S3.** [Likelihood ratio test examining linearity of association between age at peak height velocity and cardiac structure and function outcomes by sex]. **Table S4.** [Distributions of imputed characteristics in the imputation datasets and in observed data (i.e. without imputation) for carotid intima-media thickness in males and females]. **Table S5.** [Distributions of imputed characteristics in the imputation datasets and in observed data (i.e. without imputation) for left ventricular mass index in males and females]. **Table S6.** [Distributions of imputed characteristics in the imputation datasets and in observed data (i.e. without imputation) for relative wall thickness in males and females]. **Table S7.** [Distributions of imputed characteristics in the imputation datasets and in observed data (i.e. without imputation) for pulse wave velocity in males and females]. **Table S8.** [Distributions of imputed characteristics in the imputation datasets and in observed data (i.e. without imputation) for systolic blood pressure in males and females]. **Table S9.** [Adjusted associations of age at peak height velocity with measures of cardiac structure and function among participants with complete-case data on exposure, outcome and covariates]. **Table S10.** [Pearson’s correlation coefficient examining the association between age at voice breaking and age at peak height velocity in males, and age at menarche and age at peak height velocity in females].

## Data Availability

The data that support the findings of this study are available from University of Bristol but restrictions apply to the availability of these data, which were used under licence for the current study, and so are not publicly available. The study website contains details of all the data that is available through a fully searchable data dictionary and variable search tool: http://www.bristol.ac.uk/alspac/researchers/our-data/
