# Supplementary material for: Puberty timing and markers of cardiovascular structure and function at 25 years: a prospective cohort study
Source: BMC Med. 2021 Mar 25;19:78. doi: 10.1186/s12916-021-01949-y (PMC7992788; doi:10.1186/s12916-021-01949-y)
Supplement: Supplementary file 1 — Additional file 1: Table S1. [Variables used in multivariable multiple imputation models]. Table S2. [Characteristics of ALSPAC participants included in the analysis by sex and based on imputed data]. Table S3. [Likelihood ratio test examining linearity of association between age at peak height velocity and cardiac structure and function outcomes by sex]. Table S4. [Distributions of imputed characteristics in the imputation datasets and in observed data (i.e. without imputation) for carotid intima-media thickness in males and females]. Table S5. [Distributions of imputed characteristics in the imputation datasets and in observed data (i.e. without imputation) for left ventricular mass index in males and females]. Table S6. [Distributions of imputed characteristics in the imputation datasets and in observed data (i.e. without imputation) for relative wall thickness in males and females]. Table S7. [Distributions of imputed characteristics in the imputation datasets and in observed data (i.e. without imputation) for pulse wave velocity in males and females]. Table S8. [Distributions of imputed characteristics in the imputation datasets and in observed data (i.e. without imputation) for systolic blood pressure in males and females]. Table S9. [Adjusted associations of age at peak height velocity with measures of cardiac structure and function among participants with complete-case data on exposure, outcome and covariates]. Table S10. [Pearson’s correlation coefficient examining the association between age at voice breaking and age at peak height velocity in males, and age at menarche and age at peak height velocity in females]. [file 12916_2021_1949_MOESM1_ESM.docx]

**Puberty Timing and Markers of Cardiovascular Structure and Function at 25 Years: A Prospective Cohort Study**

Gillian M. Maher, Lisa Ryan, Fergus P. McCarthy, Alun Hughes, Chloe Park, Abigail Fraser, Laura Howe; Patricia M. Kearney, Linda M. O’Keeffe

**Additional file 1**

**Table S1:** Variables used in multivariable multiple imputation models.

**Table S2:** Characteristics of ALSPAC participants included in the analysis by sex and based on imputed data.

**Table S3:** Likelihood ratio test examining linearity of association between age at peak height velocity and cardiac structure and function outcomes by sex.

**Table S4:** Distributions of imputed characteristics in the imputation datasets and in observed data (i.e. without imputation) for carotid intima-media thickness in males and females.

**Table S5:** Distributions of imputed characteristics in the imputation datasets and in observed data (i.e. without imputation) for left ventricular mass index in males and females.
**Table S6:** Distributions of imputed characteristics in the imputation datasets and in observed data (i.e. without imputation) for relative wall thickness in males and females.
**Table S7:** Distributions of imputed characteristics in the imputation datasets and in observed data (i.e. without imputation) for pulse wave velocity in males and females.
 **Table S8:** Distributions of imputed characteristics in the imputation datasets and in observed data (i.e. without imputation) for systolic blood pressure in males and females.
**Table S9:** Adjusted associations of age at peak height velocity with measures of cardiac structure and function among participants with complete-case data on exposure, outcome and covariates.

**Table S10:** Pearson’s correlation coefficient examining the association between age at voice breaking and age at peak height velocity in males, and age at menarche and age at peak height velocity in females.

**Table S1: Variables used in multivariable multiple imputation models**

| **Variable** | **Type of variable** | **Model used to**  **predict missing**  **data in this variable** | **How variable**  **entered when used**  **to predict missing**  **in other variables** |
| --- | --- | --- | --- |
| aPHV | Continuous | n/a, complete case was used | Continuous |
| CIMT at age 17 | Continuous | Linear regression | Continuous |
| CIMT at age 25 | Continuous | Linear regression | Continuous |
| LVMI at age 17 | Continuous | Linear regression | Continuous |
| LVMI at age 25 | Continuous | Linear regression | Continuous |
| RWT at age 17 | Continuous | Linear regression | Continuous |
| RWT at age 25 | Continuous | Linear regression | Continuous |
| PWV at age 17 | Continuous | Linear regression | Continuous |
| PWV at age 25 | Continuous | Linear regression | Continuous |
| SBP at age 17 | Continuous | Linear regression | Continuous |
| SBP at age 25 | Continuous | Linear regression | Continuous |
| Maternal age | Continuous | Linear regression | Continuous |
| Gestational age | Ordered categorical  (10 categories) | Ordinal logistic  regression | 9 indicator  variables |
| Household social class | Categorical  (4 categories) | Multinomial logistic  regression | 3 indicator  variables |
| Maternal education | Categorical  (4 categories) | Multinomial logistic  regression | 3 indicator  variables |
| Paternal education | Categorical  (4 categories) | Multinomial logistic  regression | 3 indicator  variables |
| Breastfeeding | Categorical  (3 categories) | Multinomial  logistic regression | 2 indicator variables |
| Birthweight | Continuous | Linear regression | Continuous |
| Maternal BMI | Continuous | Linear regression | Continuous |
| Maternal smoking | Categorical  (2 categories) | Multinomial logistic  regression | 1 indicator variable |
| Height at age 9 | Continuous | Linear regression | Continuous |
| Fat mass at age 9 | Continuous | Linear regression | Continuous |
| Parity | Categorical  (3 categories) | Multinomial  logistic regression | 2 indicator variables |
| Marital status | Categorical  (3 categories) | Multinomial  logistic regression | 2 indicator variables |
| Abbreviations: aPHV, age at peak height velocity; CIMT, carotid intima-media thickness; LVMI, left ventricular mass index; RWT, relative wall thickness; PWV, pulse wave velocity; SBP, systolic blood pressure; BMI, body mass index. | | | |
|  | | | |

**Table S2: Characteristics of ALSPAC participants included in the analysis by sex and based on imputed data**

|  | **Males**  **N=1931** | **Females**  **N=2408** |
| --- | --- | --- |
|  | **n (%)** | **n (%)** |
| ***Household social class*** |  |  |
| Professional | 385 (19.94) | 416 (17.29) |
| Managerial/technical | 920 (47.63) | 1091 (45.29) |
| Non-manual | 414 (21.43) | 574 (23.83) |
| Manual/part skills/unskilled | 212 (11.00) | 327 (13.59) |
| ***Maternal education*** |  |  |
| Less than O level | 328 (17.01) | 432 (17.94) |
| O level | 619 (32.08) | 841 (34.92) |
| A level | 585 (30.26) | 669 (27.77) |
| Degree or above | 399 (20.65) | 466 (19.37) |
| ***Mother’s partner’s education*** |  |  |
| Less than O level | 418 (21.64) | 638 (26.49) |
| O level | 429 (22.21) | 506 (21.01) |
| A level | 560 (28.99) | 692 (28.74) |
| Degree or above | 524 (27.16) | 572 (23.76) |
| ***Breastfeeding until 3 months*** |  |  |
| Exclusively | 668 (34.57) | 919 (38.16) |
| Non-exclusively | 1034 (53.53) | 1128 (46.83) |
| Never | 229 (11.90) | 361 (15.01) |
| ***First-born child*** | 964 (49.92) | 1165 (48.37) |
| ***Maternal marital status*** |  |  |
| Never married | 232 (11.99) | 321 (13.33) |
| Married | 1609 (83.34) | 1974 (81.96) |
| Widowed/divorced/separated | 90 (4.67) | 113 (4.71) |
| ***Maternal smoking status*** |  |  |
| No | 1634 (84.62) | 2043 (84.84) |
| Yes | 297 (15.38) | 365 (15.16) |
|  | **Mean (SE)** | **Mean (SE)** |
| Maternal age at delivery (years) | 29.66 (0.10) | 29.36 (0.09) |
| Gestational age (weeks) | 39.32 (0.04) | 39.51 (0.03) |
| Birthweight (kg) | 3.49 (0.01) | 3.38 (0.01) |
| Maternal BMI (kg/m^2^) | 22.91 (0.08) | 22.74 (0.07) |
| Height at age 9 (cm) | 139.93 (0.18) | 139.02 (0.18) |
| Fat mass at age 9 (kg) | 33.92 (0.15) | 34.18 (0.15) |
| Age at peak height velocity (years) | 13.54 (0.02) | 11.73 (0.01) |
| CIMT at age 25 (mm) | 0.46 (0.001) | 0.45 (0.001) |
| LVMI at age 25 (g/m^2.7^) | 32.68 (0.28) | 29.32 (0.18) |
| RWT at age 25 | 0.36 (0.002) | 0.35 (0.001) |
| PWV at age 25 (m/s) | 6.63 (0.04) | 6.10 (0.02) |
| SBP at age 25 (mmHg) | 122.80 (0.27) | 111.66 (0.21) |

Abbreviations: SE, standard error; BMI, body mass index; CIMT, carotid intima-media thickness; LVMI, left ventricular mass index; RWT, relative wall thickness; PWV, pulse wave velocity; SBP, systolic blood pressure

**Table S3: Likelihood ratio test examining linearity of association between age at peak height velocity and cardiac structure and function outcomes by sex**

|  | **Males** | **Females** |
| --- | --- | --- |
|  | **P value comparing models** | **P value comparing models** |
| CIMT | 0.82 | 0.18 |
| LVMI | 0.39 | 0.61 |
| RWT | 0.15 | 0.52 |
| PWV | 0.33 | 0.28 |
| SBP | 0.59 | 0.73 |
|  |  |  |

P-value from likelihood ratio test comparing fit of models regressing CIMT, LVMI, RWT, PWV and SBP on fourths of aPHV (treated as a continuous exposure) to models regressing CIMT, LVMI, RWT, PWV and SBP on fourths of aPHV (treated as a categorical exposure).

P>0.05 indicates the more parsimonious model (aPHV treated as a continuous exposure) is a better fit, suggesting linearity of associations of aPHV and each of the outcomes.

**Table S4: Distributions of imputed characteristics in the imputation datasets (N=4,339) and in observed data (N=1199) (i.e. without imputation) for carotid intima-media thickness in males and females**

| **Imputed variable** | **% imputed data** | **Distribution**  **% for categorical variables**  **Mean (SE) for continuous variables** | | **% imputed data** | **Distribution**  **% for categorical variables**  **Mean (SE) for continuous variables** | |
| --- | --- | --- | --- | --- | --- | --- |
|  | **Males** | | | **Females** | | |
|  |  | **Imputed dataset** | **Observed data (without imputation)** |  | **Imputed dataset** | **Observed data (without imputation)** |
| ***Household social class*** |  |  |  |  |  |  |
| Professional | 10.1 | 19.94 | 25.32 | 10.2 | 17.29 | 18.90 |
| Managerial/technical |  | 47.63 | 47.47 |  | 45.29 | 47.59 |
| Non-manual |  | 21.43 | 18.57 |  | 23.83 | 24.28 |
| Manual/part skills/unskilled |  | 11.00 | 8.65 |  | 13.59 | 9.24 |
| ***Maternal education*** |  |  |  |  |  |  |
| Less than O level | 6.7 | 17.01 | 12.03 | 7.0 | 17.94 | 12.14 |
| O level |  | 32.08 | 29.75 |  | 34.92 | 37.24 |
| A level |  | 30.26 | 32.91 |  | 27.77 | 28.00 |
| Degree or above |  | 20.65 | 25.32 |  | 19.37 | 22.62 |
| ***Mother’s partner’s education*** |  |  |  |  |  |  |
| Less than O level | 8.6 | 21.64 | 17.72 | 9.2 | 26.49 | 21.38 |
| O level |  | 22.21 | 17.51 |  | 21.01 | 21.38 |
| A level |  | 28.99 | 29.75 |  | 28.74 | 28.55 |
| Degree or above |  | 27.16 | 35.02 |  | 23.76 | 28.69 |
| ***Breastfeeding until 3 months*** |  |  |  |  |  |  |
| Exclusively | 10.5 | 34.57 | 37.34 | 11.7 | 38.16 | 40.14 |
| Non-exclusively |  | 53.53 | 52.11 |  | 46.83 | 45.66 |
| Never |  | 11.90 | 10.55 |  | 15.01 | 14.21 |
| ***First-born child*** | 6.6 | 49.92 | 49.37 | 8.0 | 48.37 | 48.14 |
| ***Maternal marital status*** |  |  |  |  |  |  |
| Never married | 5.9 | 11.99 | 8.44 | 6.1 | 13.33 | 8.00 |
| Married |  | 83.34 | 87.13 |  | 81.96 | 87.45 |
| Widowed/divorced/separated |  | 4.67 | 4.43 |  | 4.71 | 4.55 |
| ***Maternal smoking status*** |  |  |  |  |  |  |
| No | 5.9 | 84.62 | 87.55 | 6.3 | 84.84 | 87.59 |
| Yes |  | 15.38 | 12.45 |  | 15.16 | 12.41 |
| Maternal age at delivery (years) | 4.6 | 29.66 (0.10) | 30.15 (0.19) | 4.8 | 29.36 (0.09) | 29.88 (0.15) |
| Gestational age (weeks) | 4.6 | 39.32 (0.04) | 39.59 (0.07) | 4.8 | 39.51 (0.03) | 39.67 (0.05) |
| Birthweight (kg) | 5.5 | 3.49 (0.01) | 3.53 (0.02) | 6.0 | 3.38 (0.01) | 3.37 (0.01) |
| Maternal BMI (kg/m^2^) | 11.5 | 22.91 (0.08) | 22.88 (0.16) | 12.7 | 22.74 (0.07) | 22.72 (0.13) |
| Height at age 9 (cm) | 4.9 | 139.93 (0.16) | 139.92 (0.27) | 5.7 | 139.02 (0.15) | 139.16 (0.22) |
| Fat mass at age 9 (kg) | 8.3 | 33.92 (0.15) | 33.66 (0.29) | 9.1 | 34.18 (0.15) | 34.04 (0.25) |
| Age at peak height velocity (years) | n/a, complete case was used | 13.54 (0.02) | 13.49 (0.04) | n/a, complete case was used | 11.73 (0.01) | 11.75 (0.03) |
| CIMT at age 25 (mm) | 66.0 | 0.46 (0.001) | 0.46 (0.002) | 56.4 | 0.45 (0.001) | 0.45 (0.001) |
| Abbreviations: BMI, body mass index; CIMT, carotid intima-media thickness. | | | | | | |

**Table S5: Distributions of imputed characteristics in the imputation datasets (N=2752) and in observed data (N=1197) (i.e. without imputation) for left ventricular mass index in males and females**

| **Imputed variable** | **% imputed data** | **Distribution**  **% for categorical variables**  **Mean (SE) for continuous variables** | | **% imputed data** | **Distribution**  **% for categorical variables**  **Mean (SE) for continuous variables** | |
| --- | --- | --- | --- | --- | --- | --- |
|  | **Males** | | | **Females** | | |
|  |  | **Imputed dataset** | **Observed data (without imputation)** |  | **Imputed dataset** | **Observed data (without imputation)** |
| ***Household social class*** |  |  |  |  |  |  |
| Professional | 9.0 | 21.56 | 23.58 | 9.1 | 18.75 | 20.08 |
| Managerial/technical |  | 47.51 | 50.53 |  | 44.58 | 48.06 |
| Non-manual |  | 20.21 | 18.11 |  | 25.13 | 23.82 |
| Manual/part skills/unskilled |  | 10.69 | 7.79 |  | 11.52 | 8.03 |
| ***Maternal education*** |  |  |  |  |  |  |
| Less than O level | 6.0 | 16.35 | 10.53 | 6.0 | 17.00 | 11.77 |
| O level |  | 31.49 | 29.89 |  | 33.88 | 34.76 |
| A level |  | 30.25 | 34.53 |  | 28.33 | 30.06 |
| Degree or above |  | 21.89 | 25.05 |  | 20.78 | 23.41 |
| ***Mother’s partner’s education*** |  |  |  |  |  |  |
| Less than O level | 7.6 | 19.31 | 15.79 | 8.0 | 23.77 | 20.23 |
| O level |  | 23.14 | 19.58 |  | 20.90 | 21.61 |
| A level |  | 28.77 | 30.74 |  | 28.53 | 27.98 |
| Degree or above |  | 28.75 | 33.89 |  | 26.77 | 30.19 |
| ***Breastfeeding until 3 months*** |  |  |  |  |  |  |
| Exclusively | 8.9 | 34.37 | 36.42 | 10.3 | 39.17 | 41.00 |
| Non-exclusively |  | 54.07 | 54.32 |  | 46.84 | 45.29 |
| Never |  | 11.55 | 9.26 |  | 13.98 | 13.71 |
| ***First-born child*** | 6.1 | 51.83 | 48.84 | 7.1 | 48.45 | 48.34 |
| ***Maternal marital status*** |  |  |  |  |  |  |
| Never married | 5.3 | 11.79 | 8.21 | 5.6 | 13.13 | 9.00 |
| Married |  | 83.22 | 87.16 |  | 82.50 | 86.57 |
| Widowed/divorced/separated |  | 4.98 | 4.63 |  | 4.35 | 4.43 |
| ***Maternal smoking status*** |  |  |  |  |  |  |
| No | 5.2 | 86.77 | 88.84 | 5.7 | 85.15 | 87.40 |
| Yes |  | 13.22 | 11.16 |  | 14.84 | 12.60 |
| Maternal age at delivery (years) | 4.2 | 29.74 (0.13) | 30.36 (0.20) | 4.3 | 29.53 (0.11) | 29.90 (0.15) |
| Gestational age (weeks) | 4.2 | 39.39 (0.05) | 39.52 (0.07) | 4.3 | 39.54 (0.04) | 39.65 (0.05) |
| Birthweight (kg) | 5.0 | 3.48 (0.01) | 3.51 (0.02) | 5.4 | 3.38 (0.01) | 3.36 (0.01) |
| Maternal BMI (kg/m^2^) | 10.4 | 22.88 (0.11) | 22.66 (0.16) | 13.0 | 22.71 (0.09) | 22.52 (0.12) |
| Height at age 9 (cm) | 4.9 | 139.77 (0.15) | 139.74 (0.27) | 5.0 | 138.94 (0.14) | 138.97 (0.22) |
| Fat mass at age 9 (kg) | 8.7 | 33.68 (0.19) | 33.08 (0.27) | 8.0 | 34.05 (0.18) | 33.76 (0.24) |
| Age at peak height velocity (years) | n/a, complete case was used | 13.51 (0.02) | 13.55 (0.04) | n/a, complete case was used | 11.75 (0.02) | 11.77 (0.03) |
| LVMI at age 25 (g/m^2.7^) | 44.4 | 32.68 (0.28) | 32.55 (0.31) | 33.2 | 29.32 (0.18) | 28.86 (0.23) |
| Abbreviations: BMI, body mass index; LVMI, left ventricular mass index. | | | | | | |

**Table S6: Distributions of imputed characteristics in the imputation datasets (N=2776) and in observed data (N=1203) (i.e. without imputation) for relative wall thickness in males and females**

| **Imputed variable** | **% imputed data** | **Distribution**  **% for categorical variables**  **Mean (SE) for continuous variables** | | **% imputed data** | **Distribution**  **% for categorical variables**  **Mean (SE) for continuous variables** | |
| --- | --- | --- | --- | --- | --- | --- |
|  | **Males** | | | **Females** | | |
|  |  | **Imputed dataset** | **Observed data (without imputation)** |  | **Imputed dataset** | **Observed data (without imputation)** |
| ***Household social class*** |  |  |  |  |  |  |
| Professional | 8.9 | 21.37 | 23.43 | 9.1 | 18.53 | 20.00 |
| Managerial/technical |  | 48.03 | 50.42 |  | 44.52 | 48.14 |
| Non-manual |  | 20.21 | 18.20 |  | 25.41 | 23.86 |
| Manual/part skills/unskilled |  | 10.37 | 7.95 |  | 11.51 | 8.00 |
| ***Maternal education*** |  |  |  |  |  |  |
| Less than O level | 5.9 | 16.32 | 10.46 | 5.9 | 17.06 | 11.86 |
| O level |  | 31.43 | 30.13 |  | 34.02 | 34.76 |
| A level |  | 30.39 | 34.52 |  | 28.22 | 30.07 |
| Degree or above |  | 21.84 | 24.90 |  | 20.69 | 23.31 |
| ***Mother’s partner’s education*** |  |  |  |  |  |  |
| Less than O level | 7.7 | 18.64 | 15.89 | 8.0 | 23.14 | 20.13 |
| O level |  | 23.00 | 19.67 |  | 20.94 | 21.52 |
| A level |  | 29.00 | 30.96 |  | 28.64 | 28.14 |
| Degree or above |  | 29.36 | 33.68 |  | 27.26 | 30.21 |
| ***Breastfeeding until 3 months*** |  |  |  |  |  |  |
| Exclusively | 9.0 | 34.61 | 36.19 | 10.2 | 38.80 | 40.97 |
| Non-exclusively |  | 53.91 | 54.60 |  | 47.13 | 45.38 |
| Never |  | 11.46 | 9.21 |  | 14.06 | 13.66 |
| ***First-born child*** | 6.2 | 52.13 | 49.16 | 7.0 | 48.72 | 48.41 |
| ***Maternal marital status*** |  |  |  |  |  |  |
| Never married | 5.2 | 11.82 | 8.16 | 5.5 | 13.17 | 9.10 |
| Married |  | 83.22 | 87.24 |  | 82.31 | 86.48 |
| Widowed/divorced/separated |  | 4.94 | 4.60 |  | 4.51 | 4.41 |
| ***Maternal smoking status*** |  |  |  |  |  |  |
| No | 5.2 | 86.57 | 88.91 | 5.7 | 85.09 | 87.45 |
| Yes |  | 13.43 | 11.09 |  | 14.91 | 12.55 |
| Maternal age at delivery (years) | 4.1 | 29.75 (0.13) | 30.32 (0.20) | 4.2 | 29.52 (0.11) | 29.91 (0.15) |
| Gestational age (weeks) | 4.1 | 39.38 (0.05) | 39.52 (0.07) | 4.2 | 39.54 (0.04) | 39.64 (0.05) |
| Birthweight (kg) | 5.0 | 3.48 (0.01) | 3.51 (0.02) | 5.4 | 3.37 (0.01) | 3.37 (0.01) |
| Maternal BMI (kg/m^2^) | 10.4 | 22.89 (0.11) | 22.67 (0.16) | 12.9 | 22.72 (0.09) | 22.53 (0.12) |
| Height at age 9 (cm) | 4.8 | 139.75 (0.17) | 139.73 (0.27) | 5.1 | 138.94 (0.16) | 138.98 (0.22) |
| Fat mass at age 9 (kg) | 8.7 | 33.70 (0.19) | 33.08 (0.27) | 8.0 | 34.05 (0.18) | 33.76 (0.24) |
| Age at peak height velocity (years) | n/a, complete case was used | 13.51 (0.02) | 13.55 (0.04) | n/a, complete case was used | 11.76 (0.02) | 11.77 (0.03) |
| RWT at age 25 | 44.5 | 0.36 (0.001) | 0.36 (0.002) | 33.5 | 0.35 (0.001) | 0.35 (0.002) |
| Abbreviations: BMI, body mass index; RWT, relative wall thickness. | | | | | | |

**Table S7: Distributions of imputed characteristics in the imputation datasets (N=3964) and in observed data (N=1394) (i.e. without imputation) for pulse wave velocity in males and females**

| **Imputed variable** | **% imputed data** | **Distribution**  **% for categorical variables**  **Mean (SE) for continuous variables** | | **% imputed data** | **Distribution**  **% for categorical variables**  **Mean (SE) for continuous variables** | |
| --- | --- | --- | --- | --- | --- | --- |
|  | **Males** | | | **Females** | | |
|  |  | **Imputed dataset** | **Observed data (without imputation)** |  | **Imputed dataset** | **Observed data (without imputation)** |
| ***Household social class*** |  |  |  |  |  |  |
| Professional | 9.9 | 20.78 | 23.59 | 9.7 | 16.90 | 19.34 |
| Managerial/technical |  | 46.65 | 49.36 |  | 46.25 | 48.40 |
| Non-manual |  | 21.55 | 18.51 |  | 23.93 | 23.25 |
| Manual/part skills/unskilled |  | 10.99 | 8.53 |  | 12.90 | 9.02 |
| ***Maternal education*** |  |  |  |  |  |  |
| Less than O level | 6.7 | 16.59 | 10.89 | 6.6 | 17.60 | 12.46 |
| O level |  | 32.25 | 31.22 |  | 34.84 | 36.18 |
| A level |  | 30.78 | 32.67 |  | 28.29 | 28.94 |
| Degree or above |  | 20.37 | 25.23 |  | 19.24 | 22.42 |
| ***Mother’s partner’s education*** |  |  |  |  |  |  |
| Less than O level | 8.5 | 21.25 | 15.61 | 8.5 | 25.70 | 21.35 |
| O level |  | 22.38 | 20.15 |  | 21.04 | 20.64 |
| A level |  | 28.88 | 31.40 |  | 28.91 | 29.54 |
| Degree or above |  | 27.46 | 32.85 |  | 24.33 | 28.47 |
| ***Breastfeeding until 3 months*** |  |  |  |  |  |  |
| Exclusively | 10.3 | 33.90 | 35.39 | 11.4 | 39.22 | 40.33 |
| Non-exclusively |  | 53.68 | 53.72 |  | 46.68 | 45.91 |
| Never |  | 12.41 | 10.89 |  | 14.09 | 13.76 |
| ***First-born child*** | 6.8 | 49.83 | 50.82 | 7.6 | 48.42 | 48.64 |
| ***Maternal marital status*** |  |  |  |  |  |  |
| Never married | 6.0 | 11.54 | 8.53 | 5.9 | 12.82 | 9.37 |
| Married |  | 83.56 | 87.11 |  | 82.37 | 86.00 |
| Widowed/divorced/separated |  | 4.89 | 4.36 |  | 4.80 | 4.63 |
| ***Maternal smoking status*** |  |  |  |  |  |  |
| No | 5.8 | 84.83 | 88.57 | 6.2 | 85.15 | 87.78 |
| Yes |  | 15.16 | 11.43 |  | 14.84 | 12.22 |
| Maternal age at delivery (years) | 4.7 | 29.68 (0.11) | 30.05 (0.18) | 4.5 | 29.45 (0.09) | 29.81 (0.14) |
| Gestational age (weeks) | 4.7 | 39.31 (0.04) | 39.53 (0.06) | 4.5 | 39.52 (0.03) | 39.60 (0.05) |
| Birthweight (kg) | 5.6 | 3.48 (0.01) | 3.51 (0.02) | 5.7 | 3.38 (0.01) | 3.37 (0.01) |
| Maternal BMI (kg/m^2^) | 11.1 | 22.83 (0.09) | 22.80 (0.15) | 12.6 | 22.66 (0.07) | 22.65 (0.11) |
| Height at age 9 (cm) | 4.6 | 139.87 (0.14) | 139.85 (0.25) | 5.5 | 139.07 (0.13) | 139.17 (0.21) |
| Fat mass at age 9 (kg) | 8.1 | 33.72 (0.15) | 33.64 (0.26) | 8.9 | 34.09 (0.15) | 34.08 (0.23) |
| Age at peak height velocity (years) | n/a, complete case was used | 13.53 (0.02) | 13.50 (0.03) | n/a, complete case was used | 11.74 (0.01) | 11.76 (0.02) |
| PWV at age 25 (m/s) | 56.3 | 6.63 (0.04) | 6.72 (0.05) | 44.3 | 6.10 (0.02) | 6.12 (0.03) |
| Abbreviations: BMI, body mass index; PWV, pulse wave velocity. | | | | | | |

**Table S8: Distributions of imputed characteristics in the imputation datasets (N=4571) and in observed data (2193) (i.e. without imputation) for systolic blood pressure in males and females**

| **Imputed variable** | **% imputed data** | **Distribution**  **% for categorical variables**  **Mean (SE) for continuous variables** | | **% imputed data** | **Distribution**  **% for categorical variables**  **Mean (SE) for continuous variables** | |
| --- | --- | --- | --- | --- | --- | --- |
|  | **Males** | | | **Females** | | |
|  |  | **Imputed dataset** | **Observed data (without imputation)** |  | **Imputed dataset** | **Observed data (without imputation)** |
| ***Household social class*** |  |  |  |  |  |  |
| Professional | 10.5 | 19.85 | 23.34 | 10.3 | 17.25 | 19.83 |
| Managerial/technical |  | 47.14 | 48.82 |  | 45.40 | 46.32 |
| Non-manual |  | 22.70 | 19.84 |  | 24.18 | 23.58 |
| Manual/part skills/unskilled |  | 10.29 | 8.00 |  | 13.15 | 10.26 |
| ***Maternal education*** |  |  |  |  |  |  |
| Less than O level | 6.7 | 16.93 | 12.51 | 7.1 | 17.71 | 12.10 |
| O level |  | 33.02 | 32.02 |  | 35.31 | 35.45 |
| A level |  | 29.75 | 31.79 |  | 28.27 | 30.63 |
| Degree or above |  | 20.28 | 23.68 |  | 18.68 | 21.82 |
| ***Mother’s partner’s education*** |  |  |  |  |  |  |
| Less than O level | 8.7 | 21.58 | 17.82 | 9.2 | 26.74 | 22.21 |
| O level |  | 22.55 | 19.95 |  | 20.91 | 20.29 |
| A level |  | 28.91 | 30.21 |  | 28.49 | 29.63 |
| Degree or above |  | 26.94 | 32.02 |  | 23.83 | 27.87 |
| ***Breastfeeding until 3 months*** |  |  |  |  |  |  |
| Exclusively | 10.6 | 34.63 | 37.88 | 11.6 | 38.13 | 40.28 |
| Non-exclusively |  | 53.09 | 52.20 |  | 47.11 | 45.94 |
| Never |  | 12.27 | 9.92 |  | 14.74 | 13.78 |
| ***First-born child*** | 6.6 | 50.31 | 50.85 | 7.7 | 48.45 | 48.62 |
| ***Maternal marital status*** |  |  |  |  |  |  |
| Never married | 6.0 | 12.20 | 10.03 | 6.2 | 13.58 | 10.49 |
| Married |  | 83.24 | 86.02 |  | 81.97 | 85.91 |
| Widowed/divorced/separated |  | 4.54 | 3.95 |  | 4.43 | 3.60 |
| ***Maternal smoking status*** |  |  |  |  |  |  |
| No | 5.7 | 84.49 | 87.49 | 6.2 | 85.13 | 88.28 |
| Yes |  | 15.51 | 12.51 |  | 14.87 | 11.72 |
| Maternal age at delivery (years) | 4.5 | 29.62 (0.10) | 30.02 (0.14) | 4.7 | 29.32 (0.09) | 29.61 (0.11) |
| Gestational age (weeks) | 4.5 | 39.32 (0.04) | 39.48 (0.05) | 4.7 | 39.51 (0.03) | 39.63 (0.04) |
| Birthweight (kg) | 5.4 | 3.49 (0.01) | 3.50 (0.01) | 5.9 | 3.38 (0.01) | 3.39 (0.01) |
| Maternal BMI (kg/m^2^) | 11.4 | 22.93 (0.08) | 22.90 (0.12) | 12.7 | 22.71 (0.07) | 22.62 (0.09) |
| Height at age 9 (cm) | 5.2 | 139.98 (0.13) | 140.06 (0.20) | 5.8 | 139.05 (0.13) | 139.03 (0.17) |
| Fat mass at age 9 (kg) | 8.3 | 34.04 (0.15) | 33.94 (0.22) | 8.9 | 34.20 (0.15) | 34.03 (0.19) |
| Age at peak height velocity (years) | n/a, complete case was used | 13.53 (0.02) | 13.48 (0.03) | n/a, complete case was used | 11.74 (0.01) | 11.75 (0.02) |
| SBP at age 25 (mmHg) | 38.1 | 122.80 (0.27) | 123.10 (0.36) | 24.8 | 111.66 (0.21) | 111.76 (0.27) |
| Abbreviations: BMI, body mass index; SBP, systolic blood pressure. | | | | | | |

**Table S9: Adjusted associations of age at peak height velocity with measures of cardiac structure and function among participants with complete-case data on exposure, outcome and covariates**

|  | **Males** | **Females** |
| --- | --- | --- |
| **Outcome** | **Estimate (95% CI)^a,b^** | **Estimate (95% CI)^a,b^** |
| **CIMT** | 0.006 (0.001, 0.01) | -0.002 (-0.007, 0.001) |
| **LVMI** | -0.18 (-0.87, 0.50) | -1.53 (-0.90, -2.16) |
| **RWT** | -0.002 (-0.008, 0.003) | -0.006 (-0.0001, -0.01) |
| **PWV** | 0.02 (-0.09, 0.14) | 0.04 (-0.05, 0.13) |
| **SBP** | 0.25 (-0.62, 1.14) | 0.01 (-0.72, 0.76) |
| ^a^Association of a one-year older aPHV with each outcome.  ^b^Adjusted for maternal age, gestational age, household social class, maternal education, mother’s partner’s education, breastfeeding of baby until three months, parity, birthweight, maternal body mass index, maternal marital status, maternal smoking status during first 3 months of pregnancy, and height and fat mass of offspring at age 9.  Abbreviations: aPHV, age at peak height velocity; CI, confidence interval; CIMT, carotid intima-media thickness; LVMI, left ventricular mass index; RWT, relative wall thickness; PWV, pulse wave velocity; SBP, systolic blood pressure. | | |

|  | **Males** | **Females** |
| --- | --- | --- |
|  | Age at voice breaking | Age at menarche |
| Age at peak height velocity | 0.315 | 0.791 |
| Results of the Pearson’s correlation coefficient between age at voice breaking and aPHV in males, and age at menarche and aPHV in females was 0.315 and 0.791 respectively indicating a moderate correlation in males and a strong correlation in females. Growth spurt and age at voice breaking in males usually occur in late puberty. Therefore, the moderate correlation observed in males is likely due to a reliance on self-reported measures of voice change, leading to measurement error^(5)^. | | |

**Table S10: Pearson’s correlation coefficient examining the association between age at voice breaking and age at peak height velocity in males, and age at menarche and age at peak height velocity in females**
